# The Thomsen-Friedenreich Antigen-Specific Antibody Signatures in Patients with Breast Cancer

**DOI:** 10.1155/2018/9579828

**Published:** 2018-07-15

**Authors:** Oleg Kurtenkov, Kaire Innos, Boris Sergejev, Kersti Klaamas

**Affiliations:** ^1^Department of Oncology and Immunology, National Institute for Health Development, Hiiu 42, 11619 Tallinn, Estonia; ^2^Department of Epidemiology and Biostatistics, National Institute for Health Development, Hiiu 42, 11619 Tallinn, Estonia

## Abstract

Alterations in the glycosylation of serum total immunoglobulins show these antibodies to have a diagnostic potential for cancer but the disease-related Abs to the tumor-associated antigens, including glycans, have still poorly been investigated in this respect. We analysed serum samples from patients with breast carcinoma (n = 196) and controls (n = 64) for the level of Thomsen-Friedenreich (TF) antigen-specific antibody isotypes, their sialylation, interrelationships, and the avidity by using ELISA with the synthetic TF-polyacrylamide conjugate as an antigen and the sialic acid-specific* Sambucus nigra* agglutinin (SNA) and ammonium thiocyanate as a chaotrope. An increased sialylation of IgG and IgM, but a lower SNA reactivity of IgA TF antibodies, and a higher level and avidity of the TF-specific IgA were found in cancer patients. Other cancer-related signatures were the highly significant increase of the IgG/IgA ratio and the very low SNA/IgA index in cancer, including patients with an early stage of the disease. These changes showed a good diagnostic potential with about 80% accuracy. Thus, the level of naturally occurring anti-TF antigen antibodies, their sialylation profile, isotype distribution, and avidity displayed cancer-specific changes that could serve as novel noninvasive Ab-based biomarkers for early breast cancer.

## 1. Introduction

The altered glycosylation often observed in cancer cells leads to the expression of modified glycopeptide epitopes, as well as tumor-associated glycans (TAG) that may be autoimmunogenic and recognized by autoantibodies [1–8]. A broad spectrum of natural and adaptive anti-glycan Abs is present in human serum in health and disease, showing a rather stable level over time in healthy people [2, 4, 9–12]. There is strong evidence that a majority of them is a result of the innate and adaptive immune response to microbial carbohydrates [13–15].

The immunoreactive Thomsen-Friedenreich glycoantigen, TF, CD176 (Gal*β*1-3GalNAc*α*-O-Ser/Thr (Core 1) structure) is expressed in about 90% of all human carcinomas but not in healthy tissues [2, 16]. The level of naturally occurring TF-specific Abs is usually decreased in cancer and is associated with tumor progression and patient survival [9, 17–19], suggesting the important role of anti-TF Abs in tumor immunosurveillance. Both murine and humanized MAbs to TF showed in vitro and in vivo activity towards TF-positive human breast cancer cell lines and in a human breast cancer xenograft model in SCID mice [20].

Immunoglobulins (Igs) are glycosylated molecules and it is now clear that the N-glycans of the Fc-fragment strongly influence IgG-Fc*γ* receptor interactions and thus the Fc-mediated effector mechanisms [21, 22]. Several studies have demonstrated that agalactosylated IgGs show an increased inflammatory activity, whereas sialylated Abs display an anti-inflammatory effect [23–25].

Compared to healthy individuals, there is a marked change of serum IgG glycosylation in individuals with autoimmune diseases, infections, and tumors [26–29], including breast cancer [29, 30]. The serum IgG glycosylation profiling has showed a diagnostic and prognostic potential in various malignancies [27, 31], including breast [30, 32]. However, it is important to note that the total serum IgG glycosylation may significantly differ from that of antigen-specific Abs [28], suggesting the presence of disease-specific IgG changes of potential clinical importance.

The glycodiversity of Abs is now a topic of interest because of the important role of glycans in the functional behavior of Abs and a possibility of constructing Ab glycoforms with the predicted potential [33, 34]. Although it is well established that antibodies are very heterogeneous by glycosylation and functionally very limited data are available on the glycodiversity of Abs to tumor-associated antigens, including TAG and of the currently used cancer biomarkers, only a few studies have been reported on the analysis of disease-specific anti-TAG Abs polymorphism, including glycosylation [35–37].

We recently established the increased *α*2,6 sialylation of TF-specific Abs in patients with gastric and colon cancer [36, and unpublished]. Moreover, some changes showed a good diagnostic potential and association with long-term survival in patients. However, it remains unclear whether this is characteristic of only gastrointestinal cancer. In the present study, we show that the levels of anti-TF antigen Abs, sialylation profile, isotypes distribution, and avidity reveal cancer-specific changes also in patients with breast cancer and can serve as diagnostic biomarkers.

## 2. Material and Methods

### 2.1. Subjects

Serum samples were taken from patients with newly diagnosed histologically verified breast carcinoma and healthy blood donors (Table 1). The investigation was carried out in accordance with the ICH GCP Standards and approved by Tallinn Medical Research Ethics Committee, Estonia. A written informed consent was obtained from each subject under study. Tumor staging was based on the histopathological (pTNM) classification of malignant tumors. The serum samples were stored in aliquots at −20°C until use.

### 2.2. TF-Specific Antibody Assay

The levels of anti-TF IgG, IgM, and IgA were determined by the enzyme-linked immunosorbent assay (ELISA) as described elsewhere [37] with some modifications. The plates (NUNC Maxisorp, Denmark) were coated with a synthetic TF-polyacrylamide conjugate (TF-PAA, Lectinity, Russia; 10 mol% of carbohydrate) in the carbonate buffer, pH 9.6. After the overnight incubation, triple washing and blocking with a Superblock solution (Pierce, USA) for 30 min at 25°C, the serum samples diluted 1:25 in PBS-0.05% Tween (Tw) were applied for 1.5 h at 25°C. After the subsequent washing with PBS-Tw, the level of bound anti-TF Abs was determined using the alkaline phosphatase (AP) conjugated goat anti-human IgG, IgM (Sigma, USA), or IgA (Dako, USA) and developed with p-nitrophenylphosphate disodium hexahydrate (pNPP, Sigma, USA). The absorbance values were read at 405 nm (Tecan Reader, Austria). The optical density value (OD) of control wells (blank: a Superblock solution instead of serum) was subtracted from that of Ab-coated wells and each sample was analysed in duplicate. To standardize the assay, standard serum (A) was included in each plate for IgG determination and lectin binding measurement. The interassay variations were minimized by using the correction factor (CF): CF = 1 / (standard serum A values – blank) x 100. The results were expressed in relative units (RU): RU = sample OD value x CF.

### 2.3. SNA Lectin Reactivity of TF-Specific Antibodies

The lectin reactivity of TF glycotope-specific antibodies was measured in a similar way, except that the binding of the neuraminic acid (sialic acid) specific Sambucus nigra agglutinin (SNA) to the absorbed anti-TF antibodies was determined as described earlier [37]. The biotinylated SNA (Vector Laboratories, Inc., USA) in 10 mmol/L Hepes, 0.15 mol/L NaCl, 0.1 mmol/L CaCl_2_, pH 7.5 was applied at a concentration of 5 *μ*g/mL for 1.5 h at 25°C. The bound lectin was detected with a streptavidin-AP conjugate (Dako, USA) and pNPP (Sigma, USA). The OD of control wells (no serum sample) was subtracted from that of Ab-coated wells to determine the lectin binding. Each sample was analysed in duplicate. The value of the SNA binding to all TF-specific Abs and the ratio of SNA binding to the level of TF-specific IgG, IgM, and IgA (SNA/Ig index) were determined.

### 2.4. Avidity of TF-Specific Antibodies

The avidity of anti-TF IgG, IgM, and IgA antibodies was determined by ELISA as described previously [38] with minimal changes. The plates were coated with the synthetic TF-polyacrylamide conjugate in the carbonate buffer, pH 9.6, 5 *μ*g per well. After the overnight incubation at +4°C, washing with PBS-0.05% Tw and blocking with the Superblock solution as above, the serum (diluted 1:25 in PBS-0.05% Tw) was applied for 1.5 hr at 25°C. After subsequent washing ammonium thiocyanate (NH_4_SCN) as a dissociating agent was added at a concentration of 1.25 mol/L for 15 min at +25°C. The bound antibodies were detected with the alkaline phosphatase conjugated goat anti-human IgG, IgM or IgA, and pNPP. The absorbance values were read at 405 nm. The relative avidity index (AI) was calculated for each sample and expressed as the percentage of reactivity remaining in the thiocyanate-treated wells in relation to that of untreated wells (PBS-Tw instead of chaotrope).

### 2.5. Statistical Analysis

The results were analysed using the nonparametric Mann–Whitney U test or Student's t-test, where appropriate, and the Pearson two-tailed correlation. The receiver operator characteristic (ROC) curve analysis was used to evaluate the sensitivity and specificity of changes found in colon cancer patients, as well as the accuracy of diagnostics. The respective difference between the groups was considered to be significant when P ≤ 0.05. All calculations were performed using the GraphPad Prism 5 and SPSS 15.0 software.

## 3. Results

### 3.1. Anti-TF IgG, IgM, and IgA Antibody Levels

**Figure 1:**
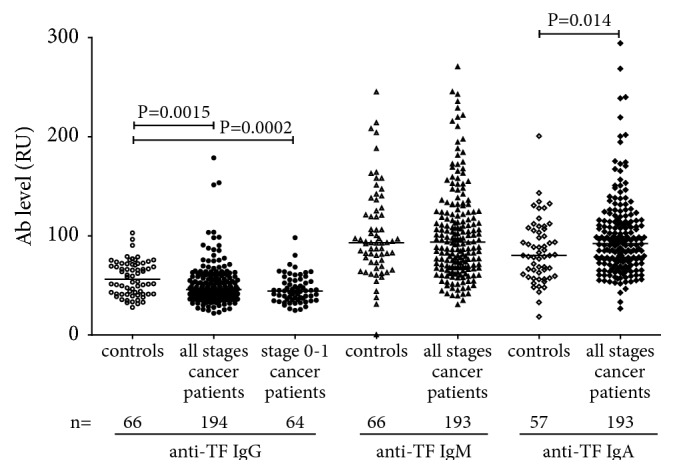
The level of TF-specific IgG, IgM, and IgA antibodies in controls and breast cancer patients. Each dot represents one individual and the group median is indicated by horizontal lines. P values were calculated by the Mann–Whitney U test and are shown for significant differences.

A significantly lower level of serum TF-specific IgG was found in cancer patients at all stages of the disease (P=0.0015), including early 0+1 stages (P=0.0002) (Figure 1).

**Figure 2:**
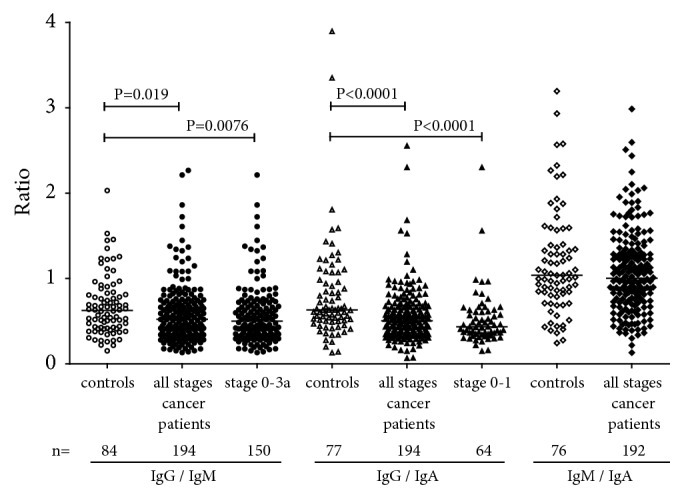
Different anti-TF antibody isotype ratios in cancer patients and controls. P values are shown for significant differences.

The anti-TF-IgM level was significantly lower only in stage 3b+3c patients (P=0.040). In contrast, an increase of the IgA Ab level was detected. No significant correlation between the levels of anti-TF antibodies of different Ig isotypes was observed in both patients and controls: IgG versus IgM, r = −0.1; IgG or IgM versus IgA r = 0.23 and 0.31 (*P*>0.05). However, the ratio IgG/IgM was significantly lower in cancer patients than in controls (P=0.019), including stages 0-3a (P=0.0076) (Figure 2). A similar decrease of IgG/IgA ratio (P <0.0001) was found in cancer patients with a more pronounced decrease at very early stages (0-1). No difference in IgM/IgA ratio (P=0.41) between patients and controls was found.

Thus, the level of some anti-TF Ab isotypes and their interrelations demonstrate significant changes in patients with breast cancer.

### 3.2. SNA Reactivity

**Figure 3:**
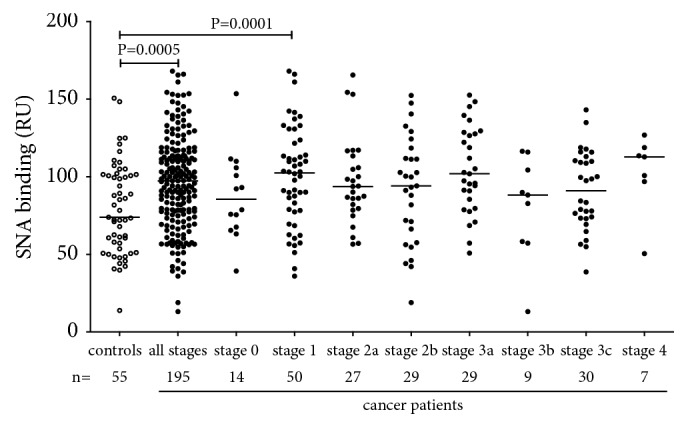
The binding of SNA lectin to TF-specific antibodies in the serum samples of cancer patients and controls. The group median is indicated by horizontal lines. P values are shown for significant differences.

A significantly higher SNA binding to anti-TF Abs (a pool of all Ig isotypes) in cancer patients compared with controls was established (P=0.0005), including stage 1 patients (P=0.001) (Figure 3).

**Figure 4:**
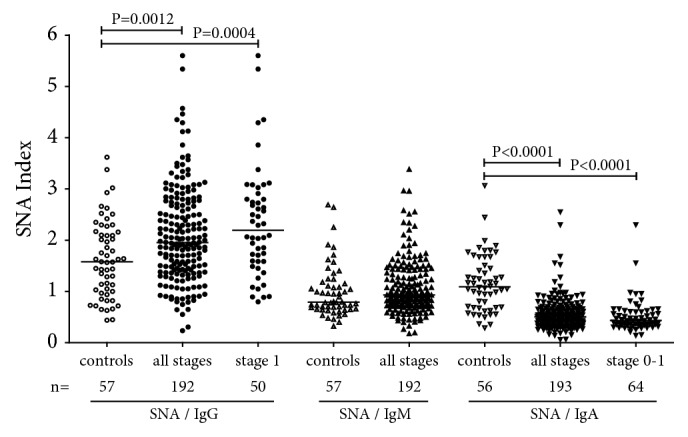
The anti-TF IgG, IgM, and IgA SNA indexes in patients and controls. P values are shown for significant differences.

The SNA/IgG index was significantly higher in cancer patients (P=0.0012) and was observed at all stages of the disease (Figure 4). In contrast, the SNA/IgA index demonstrated a marked decrease in the cancer group (<0.0001) irrespective of the disease stage especially in early cancer (P <0.0001 for stage 0+1 patients). The SNA/IgM index revealed no significant difference between patients and the controls though a slight trend to increased values was detected (p=0.12).

These findings show that all anti-TF Ab isotypes contribute to cancer-related changes of the SNA reactivity of TF-specific Abs. It appears that IgG and IgM are responsible for the increase of SNA lectin binding in cancer.

### 3.3. Avidity of Anti-TF Abs in Breast Cancer Patients and Controls

**Figure 5:**
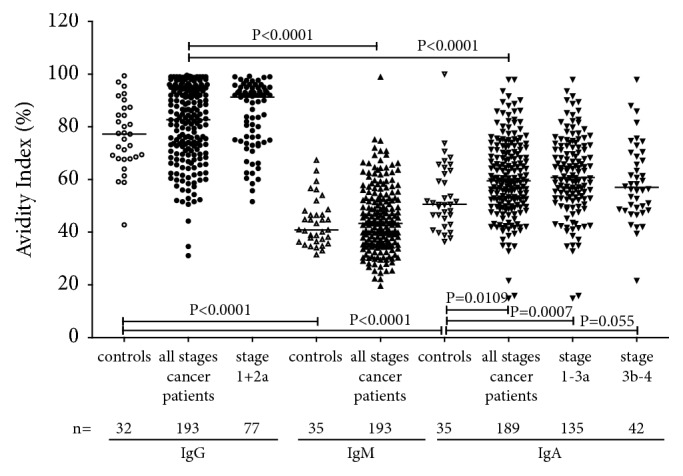
The avidity of anti-TF IgG, IgM, and IgA antibodies in controls and cancer patients.

**Figure 6:**
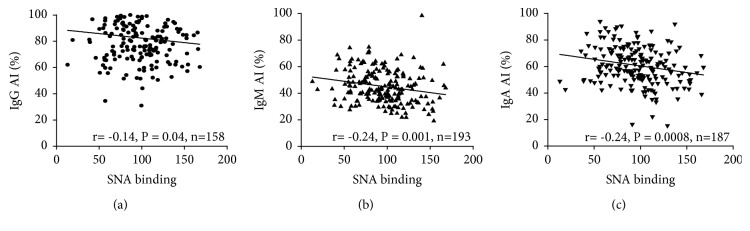
The correlation between the SNA lectin binding and the avidity of anti-TF IgG, IgM, and IgA in breast cancer patients.

No changes in the avidity of anti-TF IgG (P=0.604) and IgM (P=0.67) were found in cancer patients unlike controls, while the IgA Abs exhibited significantly higher avidity index values (P=0.0109) especially at the earlier stages of the disease ((1-3 a; P=0.0007) (Figure 5). In both cancer patients and controls, the IgG Abs showed a much higher avidity compared with IgM and IgA: P< 0.0001 in all comparisons. A significant negative correlation between the SNA binding and the avidity of anti-TF IgM, IgA and, to a lesser extent, IgG (P=0.03) was found in cancer patients (Figure 6). A similar trend was established in controls for IgM (r=-0.31, P=0.08) but not for IgG and IgA (P=0.75 and 0.23, respectively).

Thus, a higher avidity of TF-specific IgA Abs was found in breast cancer patients. An increased SNA reactivity of anti-TF antibodies in breast cancer patients was associated with the prevalence of the lower avidity TF-specific antibodies.

### 3.4. Diagnostic Potential

**Figure 7:**
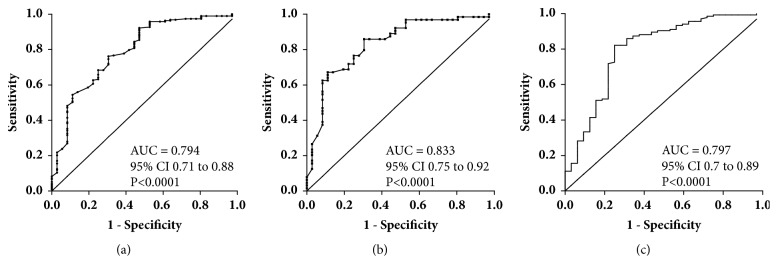
A receiver operator characteristic (ROC) curve analysis for anti-TF IgA-related parameters// SNA/IgA index and the avidity of IgA. (a) SNA/IgA index for all cancer patients; (b) SNA/IgA index for patients with 0-1 stage of cancer: (c) IgA avidity index for all breast cancer patients. The area under the ROC curve represents the diagnostic accuracy of changes in cancer.

The cancer-associated anti-TF Ab diversity differences were analysed by the Receiver Operator Curve (ROC) analysis to assess their possible potential for cancer-noncancer group discrimination (Table 2, Figure 7).

More informative data were noted about the highly significant decrease of the SNA/IgA index, which demonstrated an about 77% accuracy of diagnostics also at the very early stages of breast cancer (0+1) (Figure 7(b)) when the sensitivity was 62.5% even at 90% specificity (Table 2). In addition, the increased avidity of anti-TF IgA Abs revealed a rather high sensitivity and specificity for cancer (75% and 82.2%, respectively, with a 80.8% accuracy of diagnostics). Despite the significant difference between patients and controls, the other parameters presented in Table 2 show diagnostic accuracy (ACC) values below 70%.

Thus, the lower SNA reactivity of anti-TF IgA antibodies (as evaluated by the SNA/IgA index), and their higher avidity demonstrated a rather good ability to discriminate patients with breast cancer from healthy controls already at the early stages of the disease.

## 4. Discussion

Unlike traditional tumor markers which are soluble proteins shed by bulky tumors, serum autoantibodies (AAbs) to TAAs are often detectable already at the early stages of cancer [38, 39]. It has been shown that the measurement of serum AAbs to a single specific TAA is usually of little value for breast cancer diagnosis [39, 40], whereas the analysis of Abs to a tailor-made panel of TAAs shows a promising diagnostic potential [41–43]. Contrary to the adaptive antibodies the naturally occurring Abs to TAA, including those to the TF antigen, are always present in the circulation, thus representing a universal and convenient target for analysis of their structural and functional alteration in neoplasia.

We proceeded from the assumption that cancer-specific signatures of anti-TFAbs may be due to their local modification by the inflammatory tumor microenvironment* in situ*. Moreover, the cancer-related changes may concern only a specific subset/glyco-subset of Abs but, at the same time, determine the main or entire functional activity and clinically important effects.

In the present study, a significant decrease of TF-specific IgG level was found already at the early stages of breast cancer. We have previously observed similar changes in patients with other cancers [9, 44, 45]. Unexpectedly, the IgA level was significantly elevated (Figure 1).

This is in contrast to our previous studies in patients with stomach, and colon cancer who showed no appreciable changes of serum IgA level [36, and unpublished] like many other natural and adaptive antibody levels in breast cancer patients [12, 39]. Notable, compared with Ab levels, the ratio between different Ab isotypes showed more pronounced differences between cancer patients, including those at the early stage of the disease (Figure 2), and controls, being highly significant for IgG/IgM and especially for IgG/IgA.

For all Ig subclasses, a low level of galactosylation and sialylation of the total serum IgG has been shown to be associated with various pathologies such as autoimmune diseases, cancer, and increased inflammation [25, 27–29, 35]. We established an increased binding of SNA to a pool of all isotypes of anti-TF Abs at all stages of cancer (Figure 3). Changes in the binding of sialic acid-specific lectin SNA to anti-TF antibodies reveal isotype-specific features. In fact, contrary to IgG and IgM, the IgA sialylation (SNA/IgA index) was very low (P<0.0001) in breast cancer patients, including stage 0-1 patients (Figure 4).

The low sialylation of TF-specific IgA Abs and their higher avidity in breast cancer patients revealed the best diagnostic potential (Table 2, Figure 7) with an about of 80% accuracy of diagnostics. We suggest that it is not the antibody level* per se* but rather the proportion of sialylated Abs among various isotypes that is more informative. Since all isotypes may compete for SNA binding, these findings need to be further specified by using purified TF-specific Ab isotypes and their ability to interact with SNA in health and cancer. The findings of the present study as well as our recent data on gastric and colon cancer support the idea that the increased sialylation of the total serum anti-TF Abs (a pool of all isotypes) is a common phenomenon in cancer despite the differences observed between various Ig isotypes. Notable, these changes are quite opposite to those found in patients with autoimmune conditions [46] where the IgG agalactosylation and asialylation are typical changes, at least for total IgG. Thus, the glycosylation profile could be an informative marker for the discrimination between these two conditions. In our opinion the antigen-specific Abs deserve more attention because their glycoprofile and functional characteristics may appreciably differ from those of total serum immunoglobulins. It has been demonstrated that the sialylation level of IgG antibodies to rheumatoid arthritis- (RA-) associated antigens but not to other IgG Abs control the arthritogenicity of RA-associated IgG [47]. Specifically, the higher sialylated IgG suppressed the development of collagen-induced arthritis. The disease-specific IgG from serum and glycopeptides attached to the IgG Fc region have been analysed by mass spectrometry and their good ability to distinguish gastric cancer from benign gastric conditions was demonstrated with a sensitivity and specificity above 80% [48]. There is evidence that the immune system drives Ab glycosylation in an antigen-specific manner [49]. Although factors contributing to the differences in the disease-specific Ab glycosylation remain not completely understood, our data support the idea that the glycoprofiling of disease-relevant autoantibodies may be a more promising way for the search of novel Ab-based biomarkers than the analysis of total serum immunoglobulins.

A general conclusion that can be drawn from our findings is that naturally occurring Abs to tumor-associated TF glycotope display cancer-specific changes that are observed already at the early stages of breast cancer. Importantly, these changes of TF-specific Abs may concern only a particular, i.e., higher sialylated subset of Abs. We suppose that the combined approach which takes into account the level of TF-specific Abs, their glycosylation profile, the relative proportions of different isotypes of Abs, their glyco-subsets, and functional characteristics has potential to be further developed into a novel noninvasive naturally occurring Ab-based methodology to cancer diagnostics and prognostics. This concept can be extended to other conditions (autoimmunity, infections) where structural and functional characterization of disease-specific Ab subsets would be of clinical importance.

## Figures and Tables

**Table 1:** The characteristics of groups under investigation.

Group	N (females)	Median age (range)
Donors	64	53 (24 - 75)

Breast cancer patients	196	62 (23 – 91)
stage 0	14	65 (29 – 82)
stage 1	50	59 (32 – 79)
stage 2a	28	59 (23 – 80)
stage 2b	29	60 (35 – 79)
stage 3a	29	58 (31 – 78)
stage 3b	9	74 (69 – 79)
stage 3c	30	67 (50 – 91)
stage 4	7	54 (38 – 71)

**Table 2:** Receiver operating characteristic (ROC) curves analysis for distinguishing cancer patients from controls.

Parameter	stage	Sensitivity	Specificity	ROC Curve Area	P Value	ACC	Sensitivity at 90%
		(95% Cl)	(95% Cl)	(95% Cl)			Specificity
IgG/IgM ratio	all	60.3% (47.2% - 72.4%)	60.8% (53.6% - 67.7%)	0.636 (55.9% - 71.4%)	0.0012	0.603	26.8%
IgG/IgM ratio	0-3a	60.3% (47.2% - 72.4%)	64.7% (56.5% - 72.3%)	0.651 (57.2% - 73.1%)	0.0005	0.620	28.7%
IgG/IgA ratio	all	60.7% (46.8% - 73.5%)	58.8% (51.5% - 65.8%)	0.636 (54.8% - 72.3%)	0.0020	0.592	17.0%
IgG/IgA ratio	0-1	66.1% (52.2% - 72.2%)	65.6% (52.7% - 77.1%)	0.690 (59.4% - 78.7%)	0.0003	0.658	21.9%
SNA binding	all	63.6% (49.5% - 76.2%)	60.5% (53.3% - 67.4%)	0.658 (57.6% - 74.0%)	0.0003	0.612	23.1%
SNA binding	1-2a, b	63.6% (49.6% - 76.2%)	61.3% (51.4% - 70.6%)	0.659 (57.1% - 74.7%)	0.0009	0.621	24.5%
SNA/IgG index	all	66.7% (49.0% - 81.4%)	65.3% (58.1% - 72.0%)	0.706 (61.0% - 80.3%)	< 0.0001	0.651	32.1%
SNA/IgG index	0+1	69.4% (51.8% - 83.7%)	70.3% (57.6% - 81.1%)	0.754 (65.2% - 85.5%)	< 0.0001	0.700	39.1%
SNA/IgA index	all	58.3% (40.8% - 74.5%)	80.3% (74.0% - 85.7%)	0.793 (71.0% - 87.7%)	< 0.0001	0.769	49.2%
SNA/IgA index	0+1	58.3% (40.8% - 74.5%)	87.5% (76.9% - 94.5%)	0.833 (74.7% - 91.8%)	< 0.0001	0.770	62.5%
IgA avidity index	1-3a	75.0% (56.6% - 88.5%)	82.2% (74.4% - 88.3%)	79.7% (70.1% - 89.3%)	< 0.0001	0.808	33.3%

The diagnostic sensitivity, specificity, and accuracy for representative parameters studied at different stages of cancer. The area under the curve (AUC) with 95% confidence interval (CI), the accuracy of diagnostics (ACC), and P values are presented. AUC: the area under the receiver operator curve (ROC).

## Data Availability

Data may be available upon request through the corresponding author.

## References

[1] Springer G. F. (1984). T and Tn, general carcinoma autoantigens. *Science*.

[2] Springer G. F. (1997). Immunoreactive T and Tn epitopes in cancer diagnosis, prognosis, and immunotherapy. *Journal of Molecular Medicine*.

[3] Hakomori S. (1989). Aberrant glycosylation in tumors and tumor-associated carbohydrate antigens. *Advances in Cancer Research*.

[4] Vollmers H. P., Brändlein S. (2007). Natural antibodies and cancer. *Journal of Autoimmunity*.

[5] Abd Hamid U. M., Royle L., Saldova R. (2008). A strategy to reveal potential glycan markers from serum glycoproteins associated with breast cancer progression. *Glycobiology*.

[6] Wandall H. H., Blixt O., Tarp M. A. (2010). Cancer biomarkers defined by autoantibody signatures to aberrant O-glycopeptide epitopes. *Cancer Research*.

[7] Kobold S., Lütkens T., Cao Y., Bokemeyer C., Atanackovic D. (2010). Autoantibodies against tumor-related antigens: incidence and biologic significance. *Human Immunology*.

[8] Monzavi-Karbassi B., Pashov A., Kieber-Emmons T. (2013). Tumor-Associated Glycans and Immune Surveillance. *Vaccines*.

[9] Kurtenkov O., Klaamas K., Mensdorff-Pouilly S., Miljukhina L., Shljapnikova L., Chužmarov V. (2007). Humoral immune response to MUC1 and to the Thomsen-Friedenreich (TF) glycotope in patients with gastric cancer: relation to survival. *Acta Oncologica*.

[10] Schwartz-Albiez R. (2012). Naturally occurring antibodies directed against carbohydrate tumor antigens. *Advances in Experimental Medicine and Biology*.

[11] Bovin N. V. (2013). Natural antibodies to glycans. *Biochemistry (Moscow)*.

[12] Díaz-Zaragoza M., Hernández-Ávila R., Viedma-Rodríguez R., Arenas-Aranda D., Ostoa-Saloma P. (2015). Natural and adaptive IgM antibodies in the recognition of tumor-associated antigens of breast cancer (Review). *Oncology Reports*.

[13] Springer G. F., Tegtmeyer H. (1981). Origin of anti-Thomsen-Friedenreich (T) and Tn agglutinins in man and in white leghorn chicks. *British Journal of Haematology*.

[14] Galili U., Mandrell R. E., Hamadeh R. M., Shohet S. B., Griffiss J. M. (1988). Interaction between human natural anti-*α*-galactosyl immunoglobulin G and bacteria of the human flora. *Infection and Immunity*.

[15] Khasbiullina N. R., Bovin N. V. (2015). Hypotheses of the origin of natural antibodies: A glycobiologist's opinion. *Biochemistry (Moscow)*.

[16] Karsten U., Goletz S. (2015). What controls the expression of the core-1 (Thomsen - Friedenreich) glycotope on tumor cells?. *Biochemistry (Moscow)*.

[17] Yu L.-G. (2007). The oncofetal Thomsen-Friedenreich carbohydrate antigen in cancer progression. *Glycoconjugate Journal*.

[18] Smorodin E., Sergeyev B., Klaamas K., Chuzmarov V., Kurtenkov O. (2013). The relation of the level of serum anti-TF, -Tn and -alpha-gal IgG to survival in gastrointestinal cancer patients. *International Journal of Medical Sciences*.

[19] Smorodin E. P., Sergeyev B. L. (2016). The level of IgG antibodies reactive to TF, Tn and alpha-Gal polyacrylamide-glycoconjugates in breast cancer patients: relation to survival. *Experimental Oncology*.

[20] Tati S., Fisk J. C., Abdullah J. (2018). Corrigendum to “Humanization of JAA-F11, a Highly Specific Anti-Thomsen-Friedenreich Pancarcinoma Antibody and In Vitro Efficacy Analysis” [Neoplasia 19.9 (2017) 716-733] (S1476558617302270) (10.1016/j.neo.2017.07.001)). *Neoplasia (United States)*.

[21] Nimmerjahn F., Ravetch J. V. (2007). Antibodies, Fc receptors and cancer. *Current Opinion in Immunology*.

[22] Raju T. S. (2008). Terminal sugars of Fc glycans influence antibody effector functions of IgGs. *Current Opinion in Immunology*.

[23] Kazatchkine M. D., Kaveri S. V. (2001). Immunomodulation of autoimmune and inflammatory diseases with intravenous immune globulin. *The New England Journal of Medicine*.

[24] Kaneko Y., Nimmerjahn F., Ravetch J. V. (2006). Anti-inflammatory activity of immunoglobulin G resulting from Fc sialylation. *Science*.

[25] Böhm S., Schwab I., Lux A., Nimmerjahn F. (2012). The role of sialic acid as a modulator of the anti-inflammatory activity of IgG. *Seminars in Immunopathology*.

[26] Mehta A. S., Long R. E., Comunale M. A. (2008). Increased levels of galactose-deficient anti-Gal immunoglobulin G in the sera of hepatitis C virus-infected individuals with fibrosis and cirrhosis. *Journal of Virology*.

[27] Kodar K., Stadlmann J., Klaamas K., Sergeyev B., Kurtenkov O. (2012). Immunoglobulin G Fc N-glycan profiling in patients with gastric cancer by LC-ESI-MS: relation to tumor progression and survival. *Glycoconjugate Journal*.

[28] Shade K., Anthony R. (2013). Antibody Glycosylation and Inflammation. *Antibodies*.

[29] Ren S., Zhang Z., Xu C. (2016). Distribution of IgG galactosylation as a promising biomarker for cancer screening in multiple cancer types. *Cell Research*.

[30] Kawaguchi-Sakita N., Kaneshiro-Nakagawa K., Kawashima M. (2016). Serum immunoglobulin G Fc region N-glycosylation profiling by matrix-assisted laser desorption/ionization mass spectrometry can distinguish breast cancer patients from cancer-free controls. *Biochemical and Biophysical Research Communications*.

[31] Kodar K., Kurtenkov O., Klaamas K. (2009). The thomsen-friedenreich antigen and *α*Gal-specific human IgG glycoforms: concanavalin a reactivity and relation to survival of cancer patients. *Immunological Investigations*.

[32] Stuchlová Horynová M., Raška M., Clausen H., Novak J. (2013). Aberrant *O*-glycosylation and anti-glycan antibodies in an autoimmune disease IgA nephropathy and breast adenocarcinoma. *Cellular and Molecular Life Sciences*.

[33] Li T., DiLillo D. J., Bournazos S., Giddens J. P., Ravetch J. V., Wang L. (2017). Modulating IgG effector function by Fc glycan engineering. *Proceedings of the National Acadamy of Sciences of the United States of America*.

[34] Yu X., Marshall M. J. E., Cragg M. S., Crispin M. (2017). Improving Antibody-Based Cancer Therapeutics Through Glycan Engineering. *BioDrugs*.

[35] Kodar K., Izotova J., Klaamas K., Sergeyev B., Järvekülg L., Kurtenkov O. (2013). Aberrant glycosylation of the anti-Thomsen-Friedenreich glycotope immunoglobulin G in gastric cancer patients. *World Journal of Gastroenterology*.

[36] Kurtenkov Oleg, Izotova Jelena, Klaamas Kersti, Sergeyev Boris (2014). Increased Sialylation of Anti-Thomsen-Friedenreich Antigen (CD176) Antibodies in Patients with Gastric Cancer: A Diagnostic and Prognostic Potential. *BioMed Research International*.

[37] Kurtenkov Oleg, Klaamas Kersti (2017). Hidden IgG Antibodies to the Tumor-Associated Thomsen-Friedenreich Antigen in Gastric Cancer Patients: Lectin Reactivity, Avidity, and Clinical Relevance. *BioMed Research International*.

[38] Heo C.-K., Bahk Y. Y., Cho E.-W. (2012). Tumor-associated autoantibodies as diagnostic and prognostic biomarkers. *BMB Reports*.

[39] Lacombe Jérôme, Mangé Alain, Solassol Jérôme (2014). Use of Autoantibodies to Detect the Onset of Breast Cancer. *Journal of Immunology Research*.

[40] Liu W., De La Torre I. G., Gutiérrez-Rivera M. C. (2015). Detection of autoantibodies to multiple tumor-associated antigens (TAAs) in the immunodiagnosis of breast cancer. *Tumor Biology*.

[41] Zhong L., Ge K., Zu J. (2008). Autoantibodies as potential biomarkers for breast cancer. *Breast Cancer Research*.

[42] Piura E., Piura B. (2010). Autoantibodies to Tumor-Associated Antigens in Breast Carcinoma. *Journal of Oncology*.

[43] Liu Y., Liao Y., Xiang L. (2017). A panel of autoantibodies as potential early diagnostic serum biomarkers in patients with breast cancer. *International Journal of Clinical Oncology*.

[44] Kurtenkov O., Miljukhina L., Smorodin J. (1999). Natural IgM and IgG antibodies to Thomsen-Friedenreich (T) antigen in serum of patients with gastric cancer and blood donors—relation to Lewis (a,b) histo-blood group phenotype. *Acta Oncologica*.

[45] Kurtenkov O., Klaamas K., Rittenhouse-Olson K. (2005). IgG immune response to tumor-associated carbohydrate antigens (TF, Tn, *α*Gal) in patients with breast cancer: Impact of neoadjuvant chemotherapy and relation to the survival. *Experimental Oncology*.

[46] Plomp R., Ruhaak L. R., Uh H. (2017). Subclass-specific IgG glycosylation is associated with markers of inflammation and metabolic health. *Scientific Reports*.

[47] Ohmi Y., Ise W., Harazono A. (2016). Sialylation converts arthritogenic IgG into inhibitors of collagen-induced arthritis. *Nature Communications*.

[48] Zhang D., Chen B., Wang Y. (2016). Disease-specific IgG Fc N-glycosylation as personalized biomarkers to differentiate gastric cancer from benign gastric diseases. *Scientific Reports*.

[49] Mahan A. E., Jennewein M. F., Suscovich T. (2016). Antigen-Specific Antibody Glycosylation Is Regulated via Vaccination. *PLoS Pathogens*.

